# 94. Efficacy of SCY-247, a Second-generation Triterpenoid Antifungal, in Three Murine Models of Invasive Fungal Infections

**DOI:** 10.1093/ofid/ofae631.031

**Published:** 2025-01-29

**Authors:** Steve Wring, Katyna Borroto-Esoda, Nathan P Wiederhold, Ashraf S Ibrahim, David A Angulo

**Affiliations:** SCYNEXIS, Inc., Jersey City, New Jersey; SCYNEXIS, Inc., Jersey City, New Jersey; University of Texas Health San Antonio, San Antonio, TX; The Lundquist Institute at Harbor-UCLA Medical Center, Torrance, CA; SCYNEXIS, Inc., Jersey City, New Jersey

## Abstract

**Background:**

There are limited treatment options for invasive fungal infections (IFIs) with broad-spectrums of activity that can be delivered both orally and intravenously. SCY-247 is a second-generation novel triterpenoid antifungal in development. It has broad-spectrum activity covering yeasts, molds and dimorphic fungi, including azole- and echinocandin-resistant strains and can be given orally or intravenously. Recent *in vivo* murine efficacy studies were performed with SCY-247 and results from 3 studies demonstrating activity against *Candida* and Mucorales are presented.

**Table 1:**
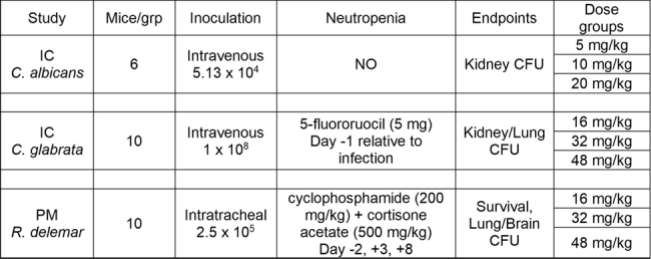
Study Methodology Details

**Methods:**

The *in vivo* activity of SCY-247 was assessed in murine models of invasive candidiasis (IC) caused by *C. albicans* and *C. glabrata* and a murine model of pulmonary mucormycosis (PM) caused by *R. delemar*. In each model, SCY-247 was administered by oral gavage, twice daily (BID), for seven days with doses ranging from 5 to 48 mg/kg. Efficacy endpoints included changes in tissue fungal burden (CFU) and/or survival. Details are shown in Table 1.

**Results:**

SCY-247 demonstrated potent *in vivo* activity in all three murine IFI models. In each dose-dependent responses based on the primary endpoints of fungal burden and/or survival were observed. Significant reductions in kidney fungal burden vs placebo (P < 0.01) were observed at doses ≥10 mg/kg against *C. albicans* and at doses ≥16 mg/kg against *C. glabrata*; significant reductions in lung fungal burden (P < 0.01) were also observed in the *C. glabrata* model at doses ≥32 mg/kg. Bioanalysis demonstrated SCY-247 preferentially distributed to kidney and lung tissues versus plasma and reductions in *C. glabrata* fungal burden correlated with dose and exposure. Against pulmonary mucormycosis, treatment with SCY-247 at doses ≥32 mg/kg resulted in prolonged survival (P < 0.05) and reductions in both lung and brain fungal burden vs placebo (P < 0.05).

**Conclusion:**

SCY-247 demonstrated significant *in vivo* activity in murine models of invasive candidiasis and pulmonary mucormycosis, and the antifungal responses correlated with dose and exposure.

**Disclosures:**

**Steve Wring, PhD**, SCYNEXIS, Inc.: Advisor/Consultant **Katyna Borroto-Esoda, MS**, Scynexis Inc: Advisor/Consultant **Nathan P. Wiederhold, PharmD**, BioMerieux: Grant/Research Support|F2G: Advisor/Consultant|F2G: Grant/Research Support|Mycovia: Grant/Research Support|Scynexis: Grant/Research Support|Sfunga: Grant/Research Support **Ashraf S. Ibrahim, PhD**, SCYNEXIS, Inc.: Advisor/Consultant|SCYNEXIS, Inc.: Grant/Research Support **David A. Angulo, MD**, SCYNEXIS, Inc.: Board Member|SCYNEXIS, Inc.: SCYNEXIS, INC Officer and Board Member, SCYNEXIS Patents|SCYNEXIS, Inc.: Stocks/Bonds (Public Company)

